# Epidemiological investigations of the introduction of porcine reproductive and respiratory syndrome virus in Chile, 2013-2015

**DOI:** 10.1371/journal.pone.0181569

**Published:** 2017-07-25

**Authors:** Víctor Neira, Barbara Brito, Juan Mena, Marie Culhane, Maria Ignacia Apel, Vanessa Max, Patricio Perez, Valentina Moreno, Christian Mathieu, Magdalena Johow, Catalina Badia, Montserrat Torremorell, Rafael Medina, Rene Ortega

**Affiliations:** 1 Departamento de Medicina Preventiva, Facultad de Ciencias Veterinarias y Pecuarias, Universidad de Chile, La Pintana, Santiago, Chile; 2 Universidad de Concepción, Facultad de Ciencias Veterinarias, Chillán, Chile; 3 College of Veterinary Medicine, University of Minnesota, St. Paul, United States of America; 4 Asociación Gremial de Productores de Cerdo de Chile, ASPROCER, Santiago, Chile; 5 Servicio Agrícola y Ganadero (SAG), División de Protección Pecuaria, Santiago, Chile; 6 Servicio Agrícola y Ganadero (SAG), Laboratorio y Estación Cuarentenaria de Lo Aguirre, Santiago, Chile; 7 Departmento de Enfermedades Infecciosas e Inmulogia Pediátrica, Escuela de Medicina, Pontificia Universidad Católica de Chile, Santiago, Chile; 8 Department of Microbiology, Icahn School of Medicine at Mount Sinai, New York, NY, United States of America; International Nutrition Inc, UNITED STATES

## Abstract

Porcine reproductive and respiratory syndrome (PRRS) is endemic in most pork producing countries. In Chile, eradication of PRRS virus (PRRSV) was successfully achieved in 2009 as a result of the combined efforts of producers and the animal health authorities. In October 2013, after several years without detecting PRRSV under surveillance activities, suspected cases were confirmed on a commercial swine farm. Here, we describe the PRRS epidemic in Chile between October 2013 and April 2015, and we studied the origins and spread of PRRSV throughout the country using official surveillance data and Bayesian phylogenetic analysis. Our results indicate that the outbreaks were caused by a PRRSV closely related to viruses present in swine farms in North America, and different from the strain that circulated in the country before 2009. Using divergence time estimation analysis, we found that the 2013–2015 PRRSV may have been circulating in Chile for at least one month before the first detection. A single strain of PRRSV spread into a limited number of commercial and backyard swine farms. New infections in commercial systems have not been reported since October 2014, and eradication is underway by clearing the disease from the few commercial and backyard farms that remain positive. This is one of the few documented experiences of PRRSV introduction into a disease-free country.

## Introduction

Porcine reproductive and respiratory syndrome (PRRS) is one of the most important viral diseases affecting domestic pigs. Clinical presentation of PRRS includes abortion, premature farrowing, stillbirths, and increased pre-weaning mortality. In growing pigs, it causes respiratory disease with decreased weight gain and increased mortality [1]. PRRS is caused by two different viral species; *PRRS virus (PRRSV) 1* and *PRRSV 2*, which were previously referred to as the European and the American PRRSV genotypes respectively. PRRS viruses are enveloped, single-stranded, positive-sense, RNA viruses, members of the family *Arteriviridae* and the genus *Porartervirus* [2].

There is broad genetic diversity within PRRSVs, which have been further grouped into 9 lineages (PRRSV 2) and 4 subtypes (PRRSV 1) based upon ORF5 phylogenetic relationships [3,4]. PRRSV 2 lineages have ~10% nucleotide differences in ORF5. Most identified and sequenced viruses of PRRSV 2 (>85%) belong to lineages 1, 5, 8 and 9 [3].

PRRSVs are endemic in many pork-producing countries worldwide and it is considered one of the most economically costly diseases for the swine industry [5]. Globally, few European countries are free of the disease, of these Sweden and Switzerland have experienced outbreaks (in 2007 and 2012 respectively) that were rapidly contained and eradication of the virus was achieved shortly after its introduction [6–8]. In South America, several countries (Argentina, Brazil, Ecuador, Paraguay, and Uruguay) have never reported PRRS disease to the World Organization for Animal Health, whereas in Colombia, Peru, Bolivia and Venezuela the virus is present [6]. Control and elimination of PRRSV is complex due to the mechanisms used by the virus to interfere with the host’s innate immune system, the ability to cause persistent infection, the spread through multiple transmission routes. Additional challenges for implementing disease control are the complex dynamics of pig movements between farms and the high biosecurity standards that must be met to avoid introduction of the virus into susceptible production systems [9].

Chile is one of the few countries that had eradicated PRRSV from its national swine population after endemic circulation. In Chile, PRRSV was first detected in 1999. Subsequently, between 2003 and 2007 a national control and eradication campaign, engaging the government and the pork industry was implemented. The eradication strategy that was implemented depended on the farm system. In farrow-to-finish farms, the eradication program consisted in total depopulation followed by repopulation with PRRSV-negative sows. In multi-site pig production systems, herd closure procedure was applied in sow farms, whereas partial or total depopulation was applied in grower or finishing farms. PRRSV was successfully eliminated from infected herds in 2009 when the last exposed animals were culled. The success of the program was attributed to the standardization and close supervision of the control protocol implementation, the limited amount of animal movement between infected herds, and the collaboration between the government and the pork industry [10]. In 2012, after 7 years of consistent surveillance activities indicating the absence of the virus, Chile declared a PRRSV-free status, which was recognized by the World Organization for Animal Health [11].

In October 2013, clinical signs compatible with PRRS were reported on a commercial swine farm located in an area of high pig density. The official Virology Laboratory at the Agriculture and Livestock Laboratories and Quarantine Stations of the Agricultural and Livestock Service (SAG), confirmed PRRSV positive samples collected from the suspected farm. Subsequent dissemination of PRRSV was reported from 45 commercial swine farms that belonged to 12 production systems (i.e. companies) and 17 backyard farms. SAG launched an official control and eradication program in May of 2014, supported by the pork producers.

In this study, we described the PRRS epidemic that affected Chile since the first positive reported farm in October 2013 until April 2015 and analyzed the evolution of PRRSV to understand its origins and spread using official outbreak data and Bayesian phylogenetic analysis. The progress of the outbreak and challenges faced while controlling the disease are discussed.

## Materials and methods

### Study area and outbreak description

#### Commercial and backyard swine in Chile

The commercial Chilean pig industry is highly integrated with approximately 190,000 sows owned by 43 pig companies that operate a total of 212 swine farms. Commercial swine farms raise pigs in confinement, containing animals in varying production stages depending on the type of farm; one-site production (farrow to finish) and multi-site production (sow farms, nursery, growing and finishing or wean to finish farm). SAG defined ‘commercial pig farms’ as those that have established biosecurity protocols and that maintain organized production records, whereas ‘non-commercial or backyard pig farms’ were defined as those without established biosecurity protocols and lacking systematically recorded production data. The definition of backyard or non-commercial pig farms was complicated by the diverse number of farms under different management practices and a varying number of animals present in the farm or household.

#### Outbreak detection

In October 2013, a commercial sow farm (breeding herd), with 2,900 sows located in Región Metropolitana (an administrative region with high pig population density), reported sudden severe clinical disease compatible with PRRS infection. As compared to the previous month, the number of live-born pigs per sow dropped from 12.5 to 7.43, and there was an increase in stillborn pigs (from 3% to 8%), mummified fetuses (from 2.2% to 33.5%) and pre-weaning mortality (from 9.2% to 67.8%; field observations). Infection was confirmed by PRRSV ELISA and rtRT-PCR two weeks after the first reported clinical case. The first reported case resulted in one isolated immediate spillover to a neighboring backyard farm through an undetermined route.

### The response of animal health authorities and the swine industry and follow-up sampling

All animals in the first detected farm (sows and suckling pigs) were culled within a month of the initial diagnosis. Sows were sent to slaughterhouses for human consumption and piglets were euthanized. However, despite this first elimination attempt, PRRSV was detected in additional farms reporting clinical disease including its nursery.

In May 2014, an official PRRS control and eradication program was launched by the SAG, which was supported by the Chilean swine industry represented by the Association of Pork Producers (ASPROCER). This program involved epidemiological investigations and active PRRSV sampling throughout the country in both, commercial and backyard pig farms to identify, reduce, contain and eliminate PRRSV from infected systems and prevent its spread to uninfected susceptible farms. Vaccination was not considered for elimination purposes and was prohibited by SAG.

Serum samples and oral fluids were collected from all commercial farms. Because of the dynamic existence of backyard farms, and the lack of official records and registries of these animals, the animal health authorities aimed at collecting serum samples from backyard farms in all administrative regions, focusing in areas surrounding commercial farms. Infected farms (cases) were defined as farms where the official SAG veterinary diagnostic laboratory confirmed the disease in at least one animal.

Samples were submitted to the SAG Virology Laboratory. Samples were centrifuged at 1,800 rpm for 5 min, and two 1 ml aliquots were made from each sample. One of the tubes was kept at the reception unit and stored at -20°C, and the other one was sent to the SAG Virology Unit, where it was stored at 4°C until further processing.

### Sample processing by real-time RT-PCR

The viral RNA was extracted using the commercial kit MagMax™ 96 Viral RNA Isolation Kit (Thermofisher, Foster City, California, USA), following the manufacturer’s instructions. The RNA was then tested by real-time RT-PCR (rtRT-PCR) using PrioCHECK® PRRSV rtRT-PCR kit (Prionics AG, Zürich, Switzerland), which can distinguish between PRRSV 1 and PRRSV 2.

### PRRS elimination from infected farms

Commercial farms identified as PRRS positive underwent a ‘herd closure’ procedure to allow the viral infection to ‘die out’ in the absence of new susceptible animals [12]. If the herd closure program was successful, the farm produced PRRSV-negative weaned pigs (by rtRT-PCR detection), but could still be positive for PRRS antibodies when assessed by serologic tests [12]. At this point (absence of viral circulation), the farms were considered ‘stable’ [13]. The stabilization of sow farms was confirmed by a 12-week follow-up protocol consisting of collecting 30 serum samples from 21 days-old pigs every two weeks (6 sampling visits total) that yielded rtRT-PCR negative results [13]. The infected grower or finishing farms were progressively depopulated, by sending animals to a slaughterhouse as they reached market weight, and not allowing the entrance of new animals into the farm until depopulation was completed.

All animals from positive backyard farms were eliminated (immediately sent to a slaughterhouse).

### Viral sequencing

PRRSV selected positive samples from different farms and with high RNA concentration (low rtRT-PCR cycle threshold value) were further processed for virus isolation and ORF5 sequencing. ORF5 codes for a structural membrane protein, it has a high genetic substitution rate and it is commonly is used to perform epidemiological investigations [14]. Virus isolation was attempted in MARC-145 (ATCC No. CRL-12231) cells monolayers as previously described [15]. For RNA nucleotide sequencing, the ORF5 was amplified using a mix of primers as previously described by Chang et al., 2002 [16]. Three ORF5 viral sequences from samples collected in Chile prior to the 2013 epidemic and stored at the SAG laboratories were also included in this study.

One hundred and forty-three samples from commercial farms independently collected by ASPROCER between October 2013 and April 2015 were sent to the University of Minnesota Veterinary Diagnostic Laboratory for viral detection and ORF5 sequencing.

One sample collected from the first reported PRRSV positive commercial farm was additionally processed to obtain the ORF7 (coding for a conserved nucleocapsid protein) nucleotide sequence [17].

### Phylogenetic analysis

ORF5 sequences from viruses collected in Chile during the outbreak and three additional sequences obtained from viruses isolated in the early 2000s in Chile were analyzed. In addition, we retrieved 61 reference PRRSV type 2 genetically diverse viral sequences from different geographic locations (North America, Asia and Europe) from GenBank to compare and reconstruct the phylogeny of the Chilean sequenced viruses. All sequences were aligned using MUSCLE [18]. The phylogeny was reconstructed using Bayesian evolutionary analysis sampling tree methods implemented in BEAST v.1.8.2 [19] software. The codon partition and substitution model were selected using Partitionfinder [20]. The analysis was run using the uncorrelated relaxed exponential clock and the coalescent Bayesian skyline population tree prior. To obtain a better resolution and parameter estimation from the phylogeny of the Chilean outbreak viruses, the analysis was repeated only with those viruses more closely related to the Chilean sequences using the same methods as described above. Convergence and mixing of the chains were assessed using Tracer [21]. The analysis was run for 5x10^8^ iterations, or until all parameters had reached an effective sample size >200 of all parameters. The final maximum clade credibility (MCC) tree was annotated and the trees sampled before convergence were burned. Time to the most recent common ancestor (tMRCA) and 95% high posterior density (HPD) estimates were obtained from the annotated tree. Bayesian phylogenetic analyses were run using computational resources available in CIPRES [22] Genetic distances of nucleotide sequences were computed using the Kimura-2 parameters substitution using MEGA v5.2.2 [23].

The ORF7 sequence obtained from an isolate from the index farm was queried using blast tools (https://blast.ncbi.nlm.nih.gov) to obtain the closest sequences available in public databases. The closest ORF7 viral sequences and additional reference sequences from all PRRS type 2 North American strains available were retrieved from GenBank to reconstruct the phylogeny and determine the viruses that were more closely related to the ones that caused the 2013–2014 Chilean outbreaks. ORF7 segments were aligned using MUSCLE, and a Maximum Likelihood phylogenetic tree was reconstructed using MEGA v5.2.2 [23].

## Results

### Epidemiological characteristic of PRRSV of the 2013 outbreak in Chile

**Fig 1:**
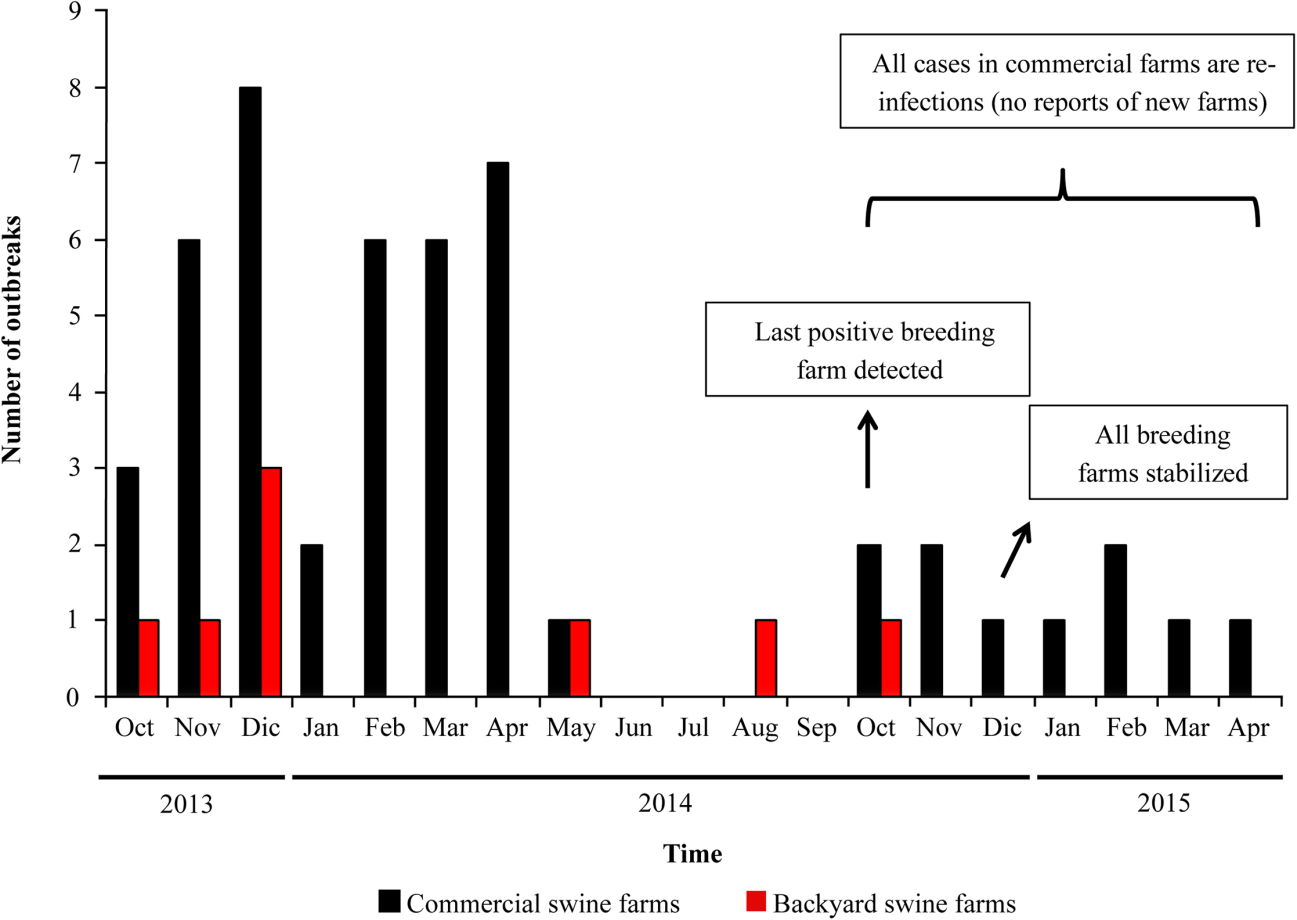
Epidemic curve showing the number of outbreaks in commercial pig farms, and backyard pigs per month from October of 2013 to April of 2015. Most new cases were reported at the beginning of the outbreak from October of 2013 to April of 2014. The last new positive breeding herd was detected in October of 2014.

From the beginning of the epidemic in October 2013 until April 2015 2, 883 pig farms including commercial and backyard were sampled throughout the country. At the beginning of the epidemic, when the number of cases peaked between October of 2013 and May of 2014, 39 commercial farms became infected (Fig 1). Between October of 2013 and April of 2015, 45 commercial and 17 backyard farms were confirmed as PRRSV positive. In 3 out of the 12 affected companies, its sow farms remained PRRSV negative. In these companies it is likely that transmissions occurred horizontally between farms rather than by internal pig flow (i.e. downstream from sow farms that supplied growing farms).

PRRSV infections in backyard pig farms were also reported during the epidemic. No further events occurred between June and July of 2014. The last new PRRSV introduction in a commercial sow farm was detected in October of 2014. Since then, all infections or reinfections correspond to growing or finishing farms, likely because of pig flows. A total of 45 swine farms had been affected by April of 2015; of which ten were sow farms; seven were nurseries; 25 growing-to-finishing; and three farrow-to-finish farms were affected. By April of 2015, 16 commercial farms remained positive, and six sow farms had been successfully stabilized.

### Genetic diversity within Chilean sequences

**Fig 2:**
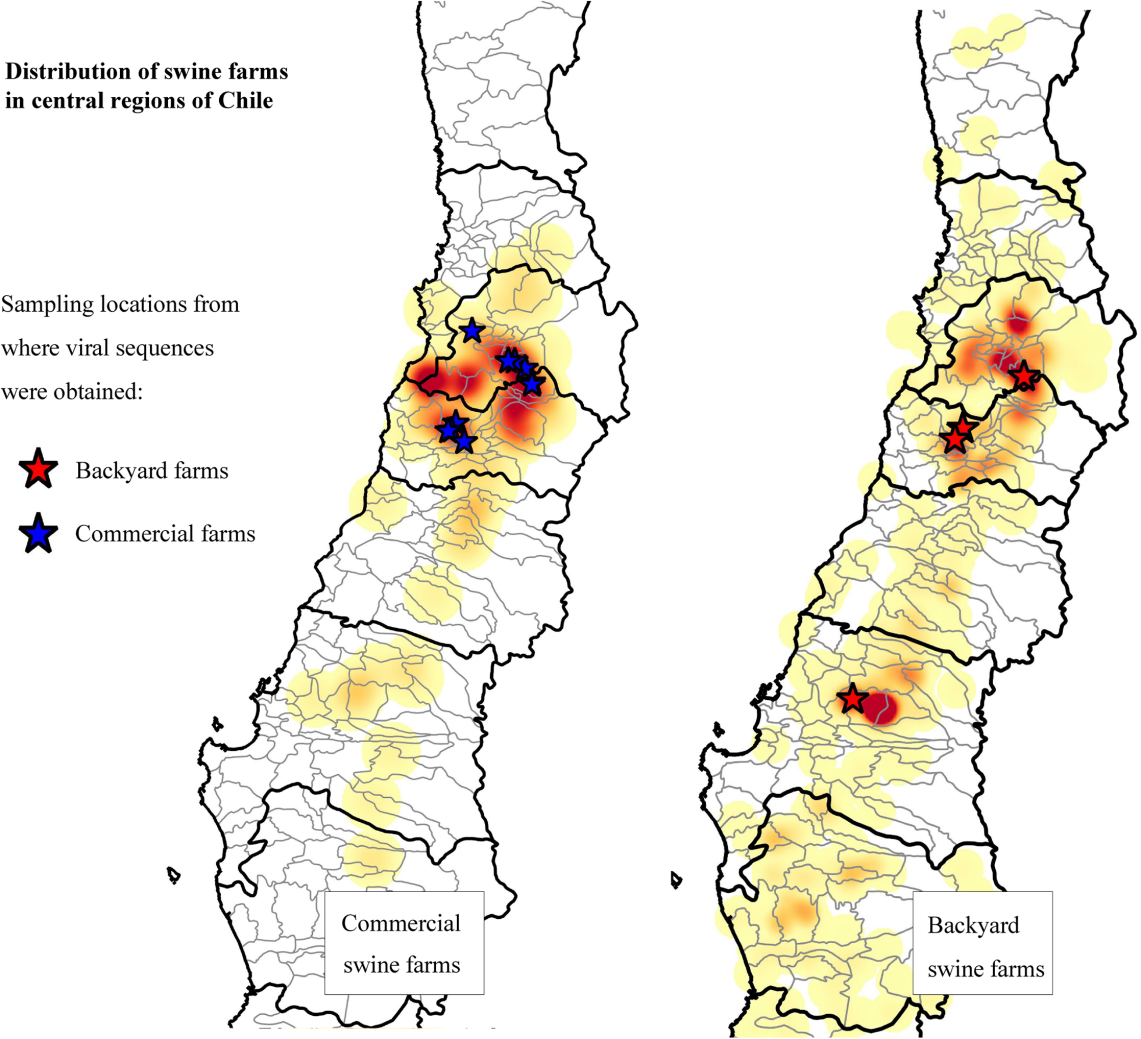
Map of sampled Chilean commercial and backyard swine during the national PRRS control and eradication program. The density of commercial and backyard farms in the central region in Chile is represented as a color gradient. Locations from where sequences were obtained are identified with stars.

All ORF5 sequences obtained were identified as North American type 2 PRRSV. The viral sequences belonged to commercial (n = 20), and backyard (n = 8) outbreak samples, and three additional sequences samples obtained in 2000, 2001 and 2006 were also included (Table 1). Fig 2 shows the location of sequenced viruses and the density of all backyard and commercial farms sampled during surveillance activities.

**Table 1:** Chilean porcine reproductive and respiratory syndrome virus sequences obtained for the study.

Sequence name	Collection date	Farm	GenBank Accession#
PRRS/5451/Chile/2013	2013/10/10	Commercial Farm 1	KX239664
PRRS/ASP1/Chile/2013	2013/10/17	Commercial Farm 1	KY972555
PRRS/ASP2/Chile/2013	2013/10/17	Commercial Farm 1	KY972556
PRRS/5659/Chile/2013	2013/10/18	Commercial Farm 1	KY972575
PRRS/5790/Chile/2013	2013/10/23	Backyard Farm 1	KY972566
PRRS/ASP3/Chile/2013	2013/10/31	Backyard Farm 1	KY972557
PRRS/ASP4/Chile/2013	2013/10/31	Backyard Farm 1	KY972558
PRRS/6494/Chile/2013	2013/11/20	Commercial Farm 2	KX239665
PRRS/ASP5/Chile/2013	2013/11/25	Commercial Farm 3	KY972559
PRRS/6631/Chile/2013	2013/11/25	Commercial Farm 3	KY972568
PRRS/6720/Chile/2013	2013/11/28	Backyard Farm 2	KY972569
PRRS/6810/Chile/2013	2013/12/03	Commercial Farm 4	KY972570
PRRS/6906/Chile/2013	2013/12/05	Commercial Farm 4	KY972571
PRRS/6913/Chile/2013	2013/12/05	Backyard Farm 3	KY972572
PRRS/6931/Chile/2013	2013/12/09	Commercial Farm 3	KX239666
PRRS/7249/Chile/2013	2013/12/20	Commercial Farm 4	KY972573
PRRS/7198/Chile/2013	2013/12/18	Backyard Farm 4	KX239667
PRRS/108/Chile/2014	2014/01/02	Commercial Farm 5	KY972574
PRRS/235/Chile/2014	2014/01/11	Commercial Farm 6	KX239668
PRRS/ASP6/Chile/2014	2014/01/27	Commercial Farm 7	KY972560
PRRS/ASP7/Chile/2014	2014/04/21	Commercial Farm 8	KY972561
PRRS/ASP8/Chile/2014	2014/04/21	Commercial Farm 8	KY972562
PRRS/2501/Chile/2014	2014/05/16	Backyard Farm 5	KX239672
PRRS/ASP9/Chile/2015	2015/04/13	Commercial Farm 10	KY972563
PRRS/ASP10/Chile/2015	2015/04/15	Commercial Farm 11	KY972564
PRRS/ASP11/Chile/2015	2015/04/16	Commercial Farm 4	KY972565
PRRS/6549/Chile/2013	2013/11/19	Commercial Farm 9	KY972567
PRRS/2483/Chile/2014	2014/05/15	Backyard Farm 5	KX239669
PRRS/2401.5/Chile/2000	2000	Commercial unidentified	KY972576
PRRS/846.2.14/Chile/2001	2001	Commercial unidentified	KY972577
PRRS/MQ.3/Chile/2006	2006	Commercial unidentified	KY972578

The genetic distance between ORF 5 PRRS Chilean sequences isolated between 2013 and 2015 ranged from 0 to 3.2x10^-2^ nucleotide substitution/site. The substitution rate of Chilean sequences analyzed by Bayesian phylogeny reconstruction was estimated at 1.0x10^-2^ (95%HPD 6.0x10^-3^–1.5x10^-2^) nucleotide substitution/site/year.

The highest identity of the Chilean PRRSV ORF5 with sequences publicly available was 97.5%, specifically from a PRRSV collected from Indiana in 2013 (PRRSV2/Indiana/XW079/2013, # KP283445). Chilean PRRSV ORF5 sequences had no indels with respect to PRRSV2/Indiana/XW079/2013. Specific amino acid differences between PRRSV2/Indiana/XW079/2013 and one of the first isolates (PRRS/5451/Chile/2013) were located at sites 15 (L->P), 58 (K->Q), 104 (K->R), 161 (I->V), and 199 (H->R). Nucleotide differences within the 28 ORF5 PRRSV sequences collected in Chile between October 2013 and April 2015, ranged from 0 to 18 (pairwise p- distance = 0.030), and the number of amino acid differences ranged from 0 to 8. Relevant initial mutations observed between the viruses collected in October 2013 and the ones collected afterward were located at amino acid sites 58 (R->Q) and 61 (N->D). Mutations observed exclusively in at least 2 of the 3 sequences collected in 2015 were located at sites: 57 (D->N), 72 (V->A), 34 (S->D), 102 (Y->H), 104 (R->G), and 106 (Y->H).

**Fig 3:**
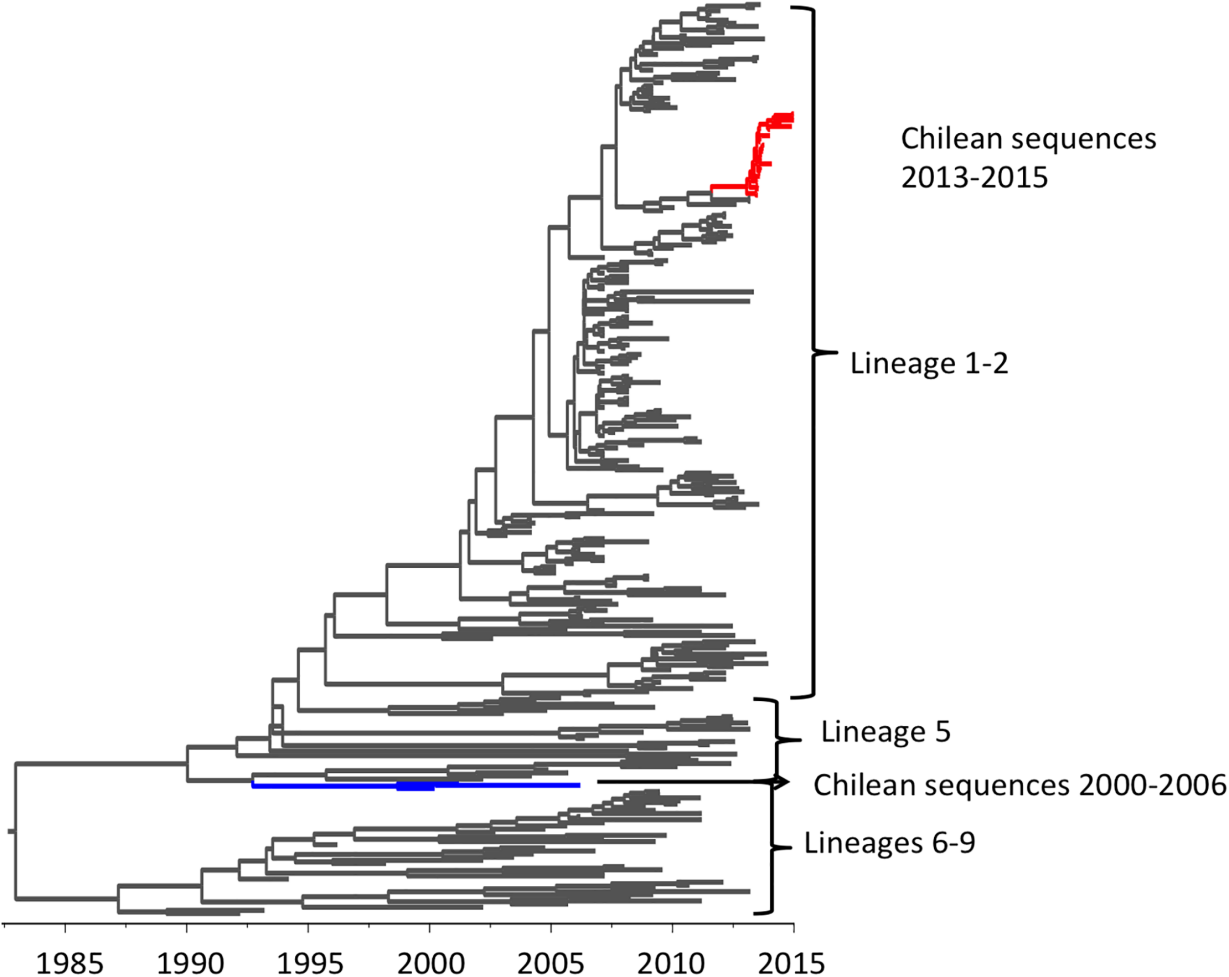
Maximum clade credibility tree of PRRSVs. Chilean isolates from 2000, 2001, and 2006 are depicted in a blue branch. Viruses from the 2013–2015 Chilean epidemic are depicted in a red branch.

The 2013–2014 Chilean and closest reference PRRSVs in reconstructed phylogeny belonged to a cluster that included viruses from lineages previously defined as 1 & 2 (Fig 3). By contrast, PRRSV sequences from viruses collected in Chile in 2000, 2001, and 2006 belonged to a monophyletic cluster, grouped with viruses of PRRSV type 2 Lineage 5 as described by Shi et al., 2010 [3] (Fig 3). ORF5 sequences from the 2013 outbreak were at least 14.33% different from the PRRSV that circulated in Chile during the early 2000’s. This finding confirms that the PRRSV that caused the 2013 outbreak was a new introduction, rather than a re-emergence of the virus previously present in the country.

**Fig 4:**
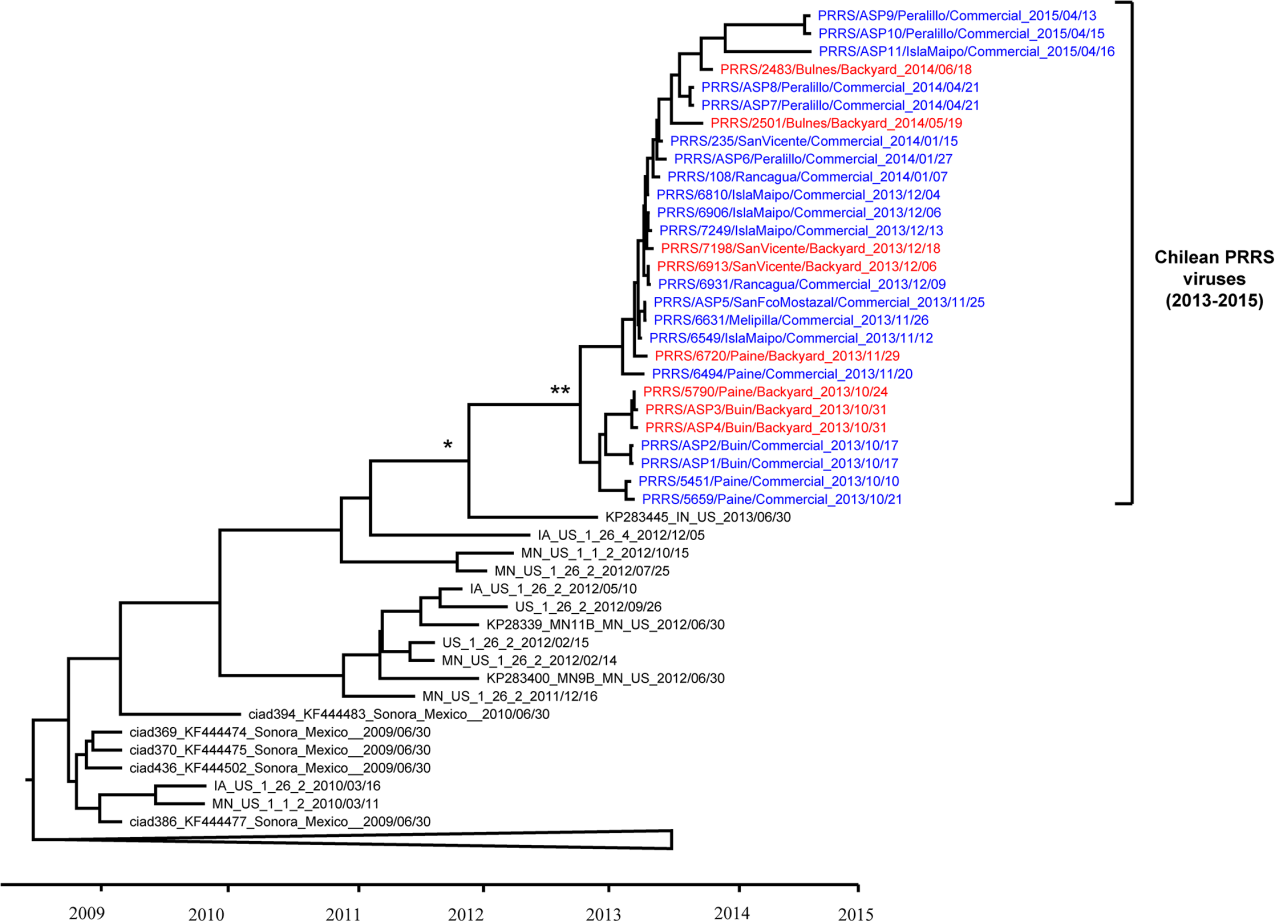
MCC phylogenetic tree of the Chilean PRRSV and closest reference sequences. The Chilean porcine reproductive and respiratory syndrome virus (PRRSV) belongs to a single monophyletic group introduced at least one month prior to the first detected case. The maximum clade credibility tree shows the Chilean PRRSV sequences collected between 2013 and 2015 and the closest reference sequences. The node showing the tMRCA of all Chilean sequences, and Chilean sequences with the closest reference PRRSV are indicated with * and ** respectively. Names of sequences sampled from commercial farms are colored in blue, and sequences from backyard pigs are indicated in red.

Viral sequences from the 2013 Chilean epidemic clustered within a monophyletic group. The tMRCA of Chilean viruses and the most closely related PRRSV reference virus (GeneBank#KP283445), which was isolated in the U.S, in 2013, was estimated in May 2012 (95%HPD April 2011-March 2013) (Fig 4). The tMRCA of all Chilean viruses collected between 2013 and 2015 was May 2013 (95%HPD October 2012-August 2013) but whether this ancestor was initially circulating on the first reported commercial farm or a different commercial or backyard farm, remains unclear. Initial branching of the 2013 Chilean viruses into two clades has a high statistical support (posterior probability). One branch comprises sequences belonging to the first reported commercial and backyard herds located nearby. This clade died out after depopulation of the first reported commercial farm (and spillover backyard farm) infected with this virus. A second clade comprised all viruses collected subsequently after the first reported case (and neighboring backyard infected farms) (Fig 4). The tree topology does not show a structured pattern of commercial and backyard samples (i.e. commercial or backyard samples grouped together in the tree), suggesting that viral circulation appears to occur between and within these two groups. However, there is low variability between viral sequences, so precise inferences of these relationships are difficult to draw.

The ORF7 was sequenced (length = 372 nucleotides) from PRRSV obtained from isolate PRRS/5451/Chile/2013 (GenBank# KY972579). A total of 85 reference viruses from all North American type 2 viruses spectrum were used to reconstruct the phylogeny. Consistent with ORF5 phylogeny, ORF7 was closely related to viruses from the United States collected between 2012 and 2014 (S1 Fig). The sequence with the highest identity (97.6%, 9 nucleotides difference) was KT257992.1 ISU73, collected from a swine farm in Missouri, 2014.

## Discussion

Results from this study show that the 2013 epidemic was caused by a single PRRSV strain genetically distinct to the one detected and eliminated from the country before 2009, confirming that the 2013 virus was a new introduction rather than a re-emergence of the virus that was present in Chile in the 2000s. Furthermore, the 2013 Chilean viruses share the closest ancestor with a contemporary North American PRRSV. Nevertheless, the reference sequences used in this study were obtained from a public repository, GenBank, and they represent only a proportion of all the variety of existing viruses, and most of them are from North America. As a consequence, more closely related viruses to the Chilean ones may not have been sequenced and published.

The introduction of PRRV into disease-free countries has been documented; however, these previous experiences differ from the Chilean epidemic in several aspects. Sweden documented PRRSV introduction in 2007. The virus was not detected by clinical disease, but by serological surveillance. Initial assessment of the situation (limited spread) allowed them to proceed with depopulation of positive premises. Retrospective inspection of production data from some the infected farms showed altered parameters such as repeat breeders and a decrease of weaned piglets. In contrast to the severe clinical disease caused by the virus introduced in Chile. From the Swedish outbreak it was concluded that the introduction may have occurred through unknown indirect contact, either from Denmark or by insufficiently disinfected sow transporting vehicles from Germany. The sequence data analysis was not informative to infer the origins because the identity with the closest ORF5 sequence available from public repositories was only 90.7% (40 nucleotide differences). Because in Europe sequencing is not used as a routine surveillance tool, its usefulness was limited in this situation [7]. By contrast, the high ORF5 sequence identity of the Chilean viruses and the North American ones allowed inference of a close relationship and elucidation of the origins of the epidemic. Another example of PRRSV introduction into a disease-free country was an outbreak that occurred in 2012 in Switzerland. The outbreak was caused by imported semen from a positive boar from Germany. The importer was alerted of the PRRSV detection, and testing of sow farms that had been inseminated with semen from the infected source as well as contact herds were conducted within a few weeks. Elimination of the virus was achieved promptly after depopulation in farms where the virus had spread [8].

The route of PRRSV introduction into Chile in 2013 is still unknown and several hypotheses have been proposed. Of these, indirect transmission of the North American PRRSV strain through neighboring countries is very unlikely because the dense-pig population areas in Chile are geographically isolated, and because cases were not located close to country borders. Other hypotheses are the importation of live animals (for genetic improvement), importation of vaccines or semen, anthropogenic (people acting as fomites), importation of swine products or illegal entry of risk materials. Introduction via animals or semen had been responsible for PRRSV transboundary transmission as mentioned above [7, 8]; however, the first reported farm did not import animals or semen, and strict health regulations restrict legal imports of animals and biological products into the country. Another hypothesis of PRRSV introduction is through imported pork, however, this route has been historically considered unlikely [24, 25]. Investigations about the different hypothesis are still ongoing.

Phylogenetic analysis of viruses circulating in commercial and backyard farms infected at the beginning of the outbreak suggests that the 2013 PRRSV had likely been circulating undetected in the Chile for at least one month prior to the reported epidemic. Therefore, although we call the first reported farm the ‘index farm’ it is also possible that the first case occurred elsewhere. Field epidemiological investigations conducted by the SAG suggest that PRRSV transmission into backyard pig farms occurs most likely as a spillover from commercial swine farms, and not in the opposite direction. This is supported by phylogenetic analysis, which shows no specific clustering of viral sequences into backyard or commercial farms, suggesting transmission between these two types of farms rather than exclusive viral circulation in any of the farm types. Spread of the disease within the country may have been related to personnel, equipment, transport as well as legal and/or illegal animal movement. Wild boars may also represent an alternative route of entry and dissemination of PRRSV. However, the presence of wild boar distribution in Chile is very limited and confined mostly to southern areas of Chile, not in the proximity of the index farm and areas with high pig population [26]. Because there are no wild boars in the northern regions of Chile, it is unlikely that there was an introduction from neighbor countries on Chile’s northern borders: Peru and Bolivia. An introduction via wild boar through southern areas of the country is also unlikely because Argentina, sharing the remaining border with Chile, has never reported PRRSV.

In this study, we used ORF5 and ORF7 to study the molecular epidemiology of PRRS in Chile. Although ORF5 and 7 cover ~7% of the PRRSV genome, it is one of the most variable regions in PRRS genome. Additionally, because ORF5 contains important antigenic sites for neutralizing antibodies, it provides relevant evolutionary information of the virus [4–27,28]. PRRSVs from the Chilean 2013 outbreak belonged to a monophyletic cluster that included viruses from lineages 1 & 2. PRRSV lineage 1 defined by Shi et al., 2010 has experienced sporadic spread throughout North America, especially between the years 2000 and 2004. In 2001, a highly pathogenic strain from this lineage, named MN184, caused severe clinical disease and spread within the US. The highly pathogenic virus showed characteristics deletions in its nsp2 [29]. One limitation of this study is that nsp2 sequences were not available for us to analyze, and therefore, we were not able to determine these markers of pathogenicity.

The number of farms infected with PRRSV has decreased gradually as a result of the National PRRSV Control and Eradication Program, and it is expected that the disease will be promptly eliminated from the country. The current eradication program is similar to the one implemented to PRRS control during the 2000s. In both programs, vaccination was forbidden. In endemic countries, PRRS vaccines have been an efficient tool to control disease by reducing clinical presentation, especially using live modified or attenuated vaccines [30–32]. However, modified live vaccines can reverse virulence, spread to other farms or can recombine with field wild-type viruses, which can originate new outbreaks [30, 33–35]. Because in Chile there was no PRRS vaccine applied, the origins of the epidemic cannot be attributed to a vaccine virus.

One challenging aspect during the Chilean epidemic was the control of reinfections in farms undergoing elimination programs. Most of these reinfections were a result of the absence of additional off-site nurseries and growing to finisher farms to perform the partial or total depopulations and difficulties in carrying out herd closure in farrow to finish farms.

Chile is one of the few countries that have achieved PRRS eradication after endemic viral circulation. However, the 2013 PRRSV introduction evidenced that despite the country’s relative isolation due to geographical barriers as well as restrictions reinforced by the national animal health services, foreign animal diseases are an important threat to the national swine population. The 2013 PRRS outbreak resulted in devastating economic losses for the industry and costly resources deployed by the animal health services for disease control. The 2013 PRRS outbreak has revealed the vulnerability of the system due to globalization and international trade. Lessons learned from this epidemic can contribute to improving prevention and emergency preparedness in PRRS-free countries as well as those areas or regions that are progressively achieving PRRS control.

## Conclusions

Results from our study show that PRRSV from the 2013–2015 epidemic in Chile was caused by a PRRSV different from the one that circulated in Chile prior to 2009. The closest reference sequences to the ones from PRRSVs that caused the Chilean epidemic were from viruses present in the United States. These PRRSVs may have been circulating in the country for at least one month before being detected. Limited transmission of the virus seems to occur between commercial and backyard farms without any structured pattern between them. Results from this study contribute to the understanding of the transboundary origin and the dynamics of transmission of PRRSV within a susceptible population in a disease-free country, and will ultimately contribute to the design of control and eradication strategies.

## Supporting information

S1 Fig Maximum Likelihood phylogenetic tree of the ORF7 obtained from the 2013–2015 epidemic in Chile. The clade (blue) with the closest viruses to the Chilean sequence (name in red) contained viruses from North America isolated in recent years.
(PDF)
