# Supplementary figures and images for: Epidemiological investigations of the introduction of porcine reproductive and respiratory syndrome virus in Chile, 2013-2015
Source: PLoS One. 2017 Jul 25;12(7):e0181569. doi: 10.1371/journal.pone.0181569 (PMC5526545; doi:10.1371/journal.pone.0181569)

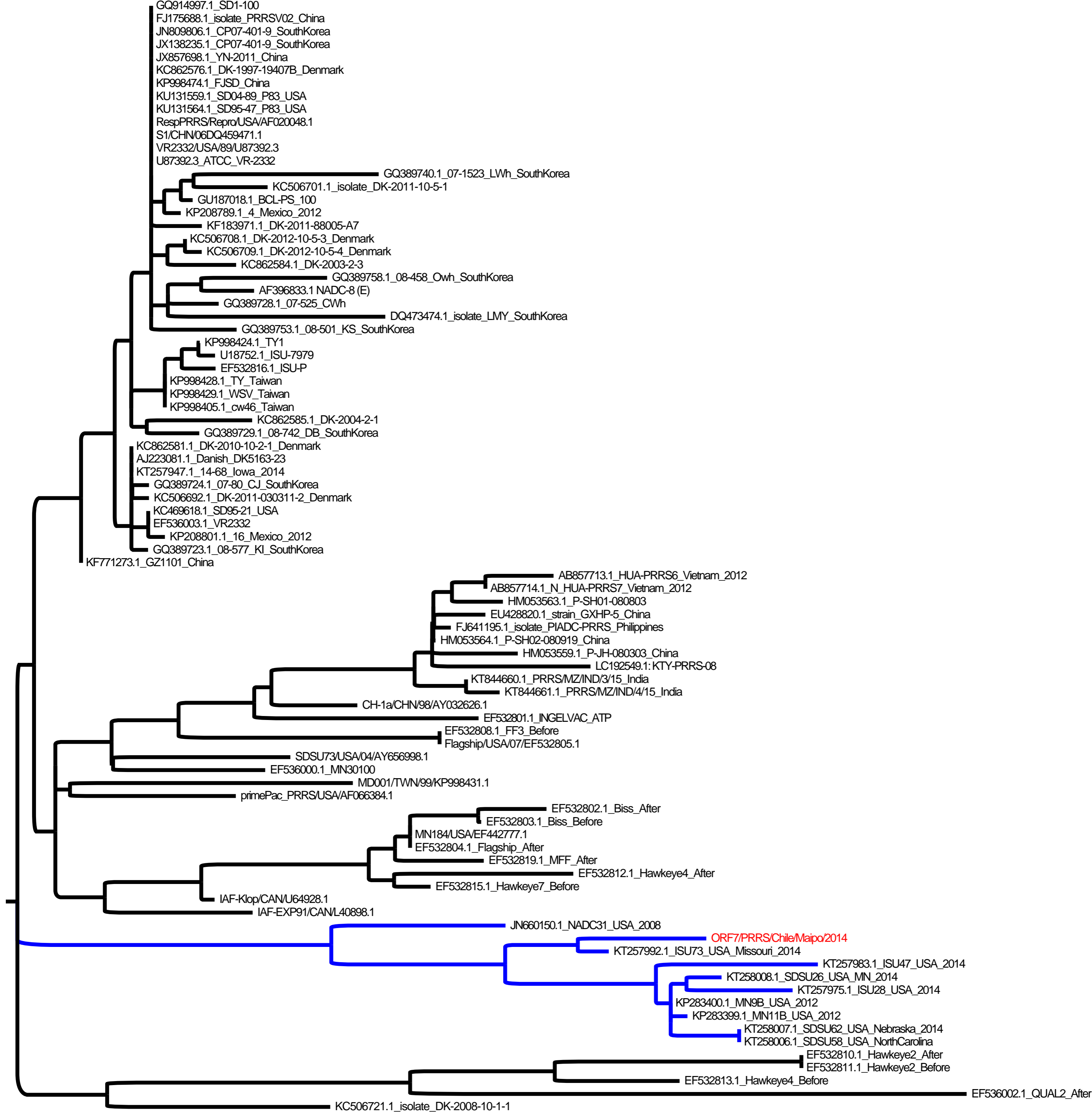

0.02

Supplement: S1 Fig — The clade (blue) with the closest viruses to the Chilean sequence (name in red) contained viruses from North America isolated in recent years. (PDF) [file pone.0181569.s001.pdf]
